# Embodied listening and coupling

**DOI:** 10.12688/f1000research.51125.1

**Published:** 2021-03-08

**Authors:** Bruno Jactat

**Affiliations:** 1 Faculty of Humanities and Social Sciences, University of Tsukuba, Tsukuba, Ibaraki, 305-8577, Japan

**Keywords:** listening, language learning, teaching, cognitive science, embodiment, situatedness, dynamic systems theory, coupling.

## Abstract

Current approaches to listening are built on standard cognitive science, which considers the brain as the locus of all cognitive activity. This work aims to investigate listening as phenomena occurring within a brain, a body (embodiment), and an environment (situatedness). Drawing on insights from physiology, acoustics, and audiology, this essay presents listening as an interdependent brain-body-environment construct grounded in dynamic systems theory.

Coupling, self-organization, and attractors are the central characteristics of dynamic systems. This article reviews the first of these aspects in order to develop a fuller understanding of how embodied listening occurs. It introduces the mind-body problem before reviewing dynamic systems theory and exploring the notion of coupling in human hearing by way of current and original analogies drawn from engineering. It posits that the current use of the Watt governor device as an analogy for coupling is too simplistic to account for the coupling phenomena in the human ear.

In light of this review of the physiological characteristics of the peripheral auditory system, coupling in hearing appears more variegated than originally thought and accounts for the diversity of perception among individuals, a cause for individual variance in how the mind emerges, which in turn affects academic performance. Understanding the constraints and affordances of the physical ear with regard to incoming sound supports the embodied listening paradigm.

## Introduction

As a second language instructor of French, I have often noted hearing difficulties among my students. I am interested in the fact that bodily functions, such as hearing, may interfere with or, conversely, help with learning a new language. Does the body actually have an influence on how the mind functions? Proponents of embodied cognition say it does and claim that the mind does not operate independently, neither from its hosts, the brain and body, nor from its environment. If this is the case, then I am curious about what a review of the human auditory system can tell us about its influence on shaping the mind.

This paper proposes that the minds of learners are impacted by the shape, size, position, fabric and functions of their physical ears, and that variability among these features accounts for diversity of minds and, by extension, variance in learning capacities. I introduce the term *embodied listening* here to refer to the fact that listening, as a cognitive trait in humans, is both constrained and facilitated by these bodily features.

While examining literature related to embodiment, I have found that some current metaphors used to describe embodied cognition are too simplistic. Dynamic systems theory is often used as a framework to explain how complex systems emerge and operate, such as embodied minds. Within this paradigm, coupling is a characteristic that defines how two separate elements such as the body and mind work in sync. An engineering device known as the Watt governor (see section *Dynamic systems theory*), is used to analogize the notion of coupling but it does so in a way that fails to capture the delicate sophistication of coupling in the hearing organ. This paper discusses these limitations and introduces the elastomer coupling device as an alternative analogy that can account for the ear’s complexity, the interactions between the different ear parts and the interactions between the environment, the physical ear and the mind.

Finally, this work is meant to provide new insights to educators who, as myself, are faced with the task of helping students learn, whether a new language or any subject that requires them to lend an ear. Ideally, it can provide us with clues as to what we can do as educators to better accommodate bodily differences.

## Topic overview

The embodied mind thesis posits that mechanisms underlying cognition are shaped not only by the brain but by the entire body. Furthermore, mind and body are not perceived as split entities. Indeed, cognitive scientists who advocate *embodiment* agree that the mind is not just operating between the ears, but that physical features of the whole body play a substantial role in the development and functioning of the mind. Shapiro refers to this as the *conceptualization* theme in embodiment: “The properties of an organism’s body limit and constrain the concepts an organism can acquire” (2019, p.4). Broadly speaking, this means, for example, that an elephant’s brain and body bring forth a mind that has little to do with the type of mind that emerges from a human brain and body. More narrowly, and more usefully, this also suggests that structural variances in the body and brain within humans generate individual differences in the mind. Since the body contributes to the overall functions of the mind through its perceptual, sensory-motor, and affective experiences with the environment, then language also, as a constituent of cognition, is equally important in the mind’s development and ongoing processing ( Lakoff *et al.*, 1999). It logically follows that academic performance is impacted by a person’s bodily mechanisms. For example, sensory processing disorders are known to cause, among other things, cognitive and academic difficulties among people on the autistic spectrum or with ADHD, and, more generally, in various degrees among people of all ages and conditions ( Geffner & Ross-Swain, 2018). As pertains to typical language learning and processing, numerous studies have reported relationships between bodily processes and language, substantializing embodied theories, notably in cognitive linguistics. In this framework, language is learned bottom-up by individuals who interact with language through their bodily and worldly experiences. Situated language use, experienced with and through a body, provides the grounding for memorizing and schematizing useful linguistic features, a process that has been well-documented by proponents of usage-based theories of language ( Tomasello, 2003; Tomasello, 2009).

If, according to the usage-based theory of language, the interactions a language user has with the environment and body matter in the building of linguistic skills, then perception must play an important role in the amount and quality of information perceived and processed. In terms of auditory perception, a review of the auditory system will shed light on its influence on learning. Research spanning fields including acoustics, audiology, anatomy, physiology, and neuroscience will prove useful to this investigation.

Since my inquiry addresses issues that are pertinent not only to researchers working in the cognitive sciences (especially language learning) but also to the boots-on-the-ground teacher trainers, language instructors, and professors, whose training might not have familiarized them with the specialized terminology associated with the fields mentioned above, I will either provide definitions for these terms or, better, will use the more common term when available (e.g. *ear canal* instead of *external auditory meatus*).

Although those scientific fields provide evidence for the embodied approach, I will include an additional framework that has often been applied in embodied research to describe the interactions between brain, body, and environment, namely dynamic systems theory. This article discusses coupling, one of the three key components of dynamic systems — coupling, self-organization, and attractors — and argues that coupling, as seen in the ear, leads to an updated understanding of listening as embodied and situated. Coupling is envisaged as the interactions between the different bone and flesh structures of the ear (or peripheral auditory system) which are schematically made up of the outer ear (ear flap, ear canal, and eardrum), middle ear (three little bones called the hammer, anvil, and stirrup) and inner ear (the cochlea, a tiny conch-like structure).

As mentioned above, some of the analogies currently used to substantiate the embodied position have proven quite attractive. However, I will also discuss some of their short-comings and provide alternatives in support of *embodied listening*.

To my knowledge, the term embodied listening has not been used in the field of cognitive linguistics. I principally use this term to refer to the fact that some of our conceptual systems, notably those related to language, are grounded in the way the physical ear processes incoming auditory inputs and subsequently, how these are processed by the higher-level auditory operations of the peripheral and central nervous systems.

By limiting this inquiry into listening to the level of perception, the present work addresses a fundamental concern in language learning: why some lower-proficiency listeners fail to process auditory information at the ascending sensory (“bottom-up”) level and thus fail in comprehension. Field explains that even if those listeners were capable of higher level (“top-down”) cognitive operations, such as inferring meaning, “they cannot employ them if they have to focus heavy attention on decoding, nor can they if there is insufficient decoded material to provide a basis for constructing meaning” (2019b, p. 309). This statement underscores the difficulties a learner is faced with when not capable of efficiently perceiving and processing aural inputs. Field’s observation also points to an area of research in language learning that needs to be addressed more substantially in order to better understand what can be done about it, namely what causes differences in decoding among learners. The present review uses the embodiment paradigm to look at how discrepancies between lower and higher proficiency listeners originate from variances in physical features.

In the end, the implications of this view of listening highlight the uniqueness of every individual’s cognitive capacities and reveal some of the reasons why each human being perceives the world differently. Ultimately, we will see how the wide variety of inclinations with which one reacts to and interacts with the world accounts for the incredible diversity of our minds.

## The mind-body split in listening

Since French mathematician, physician, and philosopher René Descartes formulated his theory of mind in 1637 ( Descartes, 1637, p.17), Western thought has been pervaded by the view that mind and body have fundamentally distinct natures and are ontologically split. On the one hand, mental properties experienced by a private self (subjectivity) include consciousness (e.g. perceptions, emotions) and intentionality (e.g. beliefs, desires). On the other hand, human physical properties observable by all (objectivity) include all matter of sizes, weights, shapes, colors, motions, etc. The relationship between these two sets of properties constitutes the mind-body problem ( Robinson, 2020). At the core of this dilemma, lies two main interrogations: what is the exact nature of these two entities (ontological question)? Also, do they influence each other and, if so, how (causal question)?

To override this conundrum, behaviorist scholars have adopted a materialist view, holding the mind to be an irrelevant abstraction to explain the rational activities of human beings. For them, mental states are but an extension of physical states. For instance, this view posits that the laws of stimulus-response alone explain the development of language ( Skinner, 1957).

Conversely, cognitivists consider the body to be irrelevant in explaining the working of the mind. Cognitivism assumes that the acquisition of mental structures arises from a determined internal matrix, a conception known as *innateness*. In the case of language, this innate matrix is referred to as the Universal Grammar, a grammar shared by all cultures and languages that arises naturally in spoken or sign language ( Pinker, 2003). In short, this theory posits that inner symbolic language (mental symbols) translates outer symbolic language (words and sentences) and thus creates meaning. These symbols are arbitrary and amodal (i.e. not linked to any particular sensory modality), and their relationships are but a network of abstract symbols that do not have physical referents to the body or the world ( De Vega *et al.*, 2012). Stated differently, abstract symbols are correlated to other abstract symbols, which are further associated with other symbols and so forth. As an example of this entanglement, imagine if someone who has never been to Japan were to ask: “What is the city of Nara like?” And, if they then received the answer: “It’s like Kyoto but greener.” Having no referent experience of being in Kyoto, this answer is wholly ineffective in helping the questioner form an image of what the city of Nara might be like. It would take a much lengthier description to provide a nebulous idea of what the former imperial capital of Nara is like ^1^. This abstract system of symbolic interactions faces a dead-end referred to as the *symbol grounding problem*. Harnad (1990) analogizes this to trying to learn Chinese with a Chinese/Chinese dictionary as the only source of available information: “The trip through the dictionary would amount to a merry-go-round, passing endlessly from one meaningless symbol […] to another […], never coming to a halt on what anything meant” (1990, p. 339). In other words, the learning would not be grounded into bodily and worldly experience. Conversely, the embodiment theory emphasizes that meaning-making in the brain is grounded in the sensorimotor experiences of the body (embodiment) interacting with the environment (situatedness), and in so doing provides a way out of the circularity of the above-mentioned symbolic system. Namely, this theory sees information given by the senses as being at the core of how we think, understand, and feel, conceiving of thought and language as the reactivation of these sensorimotor experiences as mental simulations ( Barsalou, 2008). In order to form a more precise idea of what Nara is like, a first step might be to show photographs or a video depicting it. The sensorimotor experience of looking at the photographs and talking about them or watching a travel video and hearing related sounds and explanations provides the grounding for associations made with the word “Nara.” Ultimately, the best way to know what “Nara” stands for, is to travel there and bathe in the sensorimotor sensations of the old city.

Having noted these trends, most research around consolidating the embodied approach still relies heavily on analyzing how meaning arises in the mind through methods such as debating the philosophical implications of such a view (cognitive linguistics, philosophy of mind) or scrutinizing the brain (neurosciences). For instance, new scientific findings in neurosciences have shown how listening to words activates sensorimotor areas of the cortex, a finding that demonstrates how perceptual processes such as listening are not isolated but embedded in the experience of one’s body interacting with the outside world. Thus, listening to words such as *kick*, *lick*, or *pick*, or phrases such as *press the piano pedal*, *bite the banana*, or *pick up the pen* activate the motor areas of the brain that respectively control leg, mouth, and hand movements ( Aziz-Zadeh *et al.*, 2006). Although fascinating breakthroughs of this sort have been made in neurosciences, the field has yet to produce an account of how meaning-making through listening arises via embodiment, namely the physical devices of audition, including the ear, auditory cortex, and the circuitry between them. My intention here is to examine these physical devices and the role they play in embodied listening while considering the support they give to the embodied approach more generally.

Another point I would like to draw attention to is that cognitivism, which primarily looks to information processing and symbol manipulation to explain cognition, has led many current scholars to adopt a disembodied theory of mind and language. In fields related to education, this Cartesian view of listening is widespread. Perusing current books on listening in linguistics (see Ashcraft & Tran, 2010; Brown & Brown, 2011; Buck, 2001; Cutler, 2012; Field, 2010; Field, 2019a; Field, 2019b; Flowerdew *et al.*, 1994; Flowerdew & Miller, 2005; Goh, 2014; Lynch, 2009; Nation & Newton, 2020; Richards, 2008; Rost & Wilson, 2013; Rost, 2016; Vandergrift & Goh, 2012; Wilson, 2008), one is hard-pressed to find any material on the physical nature of listening, with the exception of Rost ^2^. Listening is entirely restrained to the sphere of the mind, with not the slightest concern for its anatomical and physiological dimensions. As a consequence, even the most recent publications concerning listening skills that are targeted toward teachers, teacher trainers, language testers, and textbook authors are largely based on the cognitivist paradigm ( Bailey, 2020; Cauldwell, 2013, Cauldwell, 2018; Conti & Smith, 2019; Nemtchinova, 2020; Ockey & Wagner, 2018; Sepulveda, 2012). The overall effect is that many educators’ ideas and teaching agendas are pervaded with the belief that “since listening is cognitive in nature, there is no need to look at its physical features”. However, as I will show, exploring the workings of the human body engaged in listening will shed new light on how learners hear, listen, and construct meaning as well as provide insight into how educators can take bodily factors into account when teaching listening. Embodied listening can provide a cohesive account of auditory perception, processing skills, and meaning-making skills and help bridge the Cartesian divide. The scope of this article only allows for a limited investigation, and I will consequently confine this study to the exploration of the peripheral auditory system. A more exhaustive examination of this perspective by way of exploring, for example, the central auditory system (e.g. auditory nerve, brainstem and brain), an endeavor I intend to achieve in a sequel article, will further substantiate the cause for embodied listening.

Before continuing, it is worth recalling the distinction between hearing and listening. Hearing refers to the reception of sounds from the open-field (e.g. speech, music, natural sounds, traffic, etc.) or emanating from within the body (e.g. digestion, the heartbeat, or disabling signals such as tinnitus). On the other hand, listening refers to higher-order cognitive processes including attention, interpretation, and response. Succinctly, hearing is “a process of perceiving sound,” while listening is “a process of making sense of those sounds for the purpose of communicative action” ( Bodie & Wolvin, 2020, p.295), and, we should add, *for the purpose of learning and making sense of the world*. In order to focus on embodied listening by exploring the ear’s functions, I will limit the upcoming review and discussion to the anatomical and physiological aspects of the peripheral auditory system (the external and middle ear). This review is grounded in the hearing processes of perception, which form the foundation of the higher processes of listening and are a crucial part of the rich interplay of operations that go into meaning-making.

## Dynamic systems theory

One framework often used to understand embodied cognition is dynamic systems theory. Thelen and Smith provide a helpful summary: “The term dynamic systems, in its most generic form, means systems of elements that change over time. The more technical use, dynamical systems, refers to a class of mathematical equations that describe time-based systems with particular properties.” (2007). This framework has often been applied to a variety of fields as a metatheory, used for describing living and nonliving systems that exhibit continuous change and re-organization over time. Applications have been found in fields such as in meteorology, for explaining cloud formation, or in biology, for, among other things, characterizing behavior in ant colonies ( Gordon, 2010). Dynamic systems theory has proven a robust theoretical framework to study the processes underlying transformations in complex systems. These changes are difficult to model because they result from interactions between the diverse elements that make up a given system/organism, whether it concerns a single system/organism (i.e. a cell) or multiple systems/organisms (i.e. our galaxy). When observing atmospheric physics or insect behavior, we can see that, despite the seemingly chaotic interactions of the constituents of a given system, organized patterns emerge in the overall system. Understanding complex systems as dynamic systems will also serve our investigation into cognitive science ( Spivey, 2008; Thelen & Smith, 2007).

Among the various central characteristics of dynamic systems, three are observable in cognitive science, thus supporting the claim that cognition is embodied and situated: the phenomenon of coupling (or interdependent cycles of causation), the self-organizing principle, and the attractors theory. I draw from the theory of coupling to provide evidence for the claim that listening, as a constituent of cognition, is naturally embodied and situated.

## Coupling

The definition of coupling found in Merriam-Webster’s online dictionary reads as follows:

1. the act of bringing or coming together: pairing. Specifically: sexual union;
2. a device that serves to connect the ends of adjacent parts or objects;
3. the joining of or the part of the body that joins the hindquarters to the forequarters of a quadruped;
4. a means of electric connection of two electric circuits by having a part common to both ^3^.

This fundamental idea of two constituent parts being connected to work together has been further developed in dynamic systems theory. In terms of cognitive science, which observes and tries to describe the complex interactions underlying the workings of the mind, the idea of coupling is meant to characterize how two systems, such as the brain and body or the body and environment, for instance, interact. A brief review of how this notion first emerged will help us grasp the point made by the proponents of embodiment and situatedness.

The phenomenon of coupling was first noted in 1665 by Dutch astronomer and physicist Christiaan Huygens (1673), who observed that two of his pendulum clocks, hung next to each other, synchronized into coupled oscillation after about 30 minutes ( Strogatz, 2003, p.106–109). The motion of the two pendulums converged until they swung with the same period and amplitude but in opposite directions. Huygens discovered the mechanism responsible for the sympathetic motion of the pendulums: the small vibrations of the wooden beam on which the clocks were hanging. The behavior of Huygens’ pendulums can be captured in a mathematical formula, in which the behavior of one pendulum also includes a term that describes the behavior of the other pendulum, a basic tenet of dynamical systems theory. Coupling might then be observed in the motions of any dyad that demonstrates such properties: two metronomes sitting on a board ( Pantaleone, 2002), the mating flashes of fireflies ( Buck & Buck, 1966), or even your own footsteps falling in step with others, a phenomenon which the fictional Welton Academy English teacher John Keating demonstrated to his pupils in the movie *Dead Poets Society* when he had his students walk in a circle around the classroom until they all unwittingly began marching in sync ( Weir, 1989).

**Figure 1.**
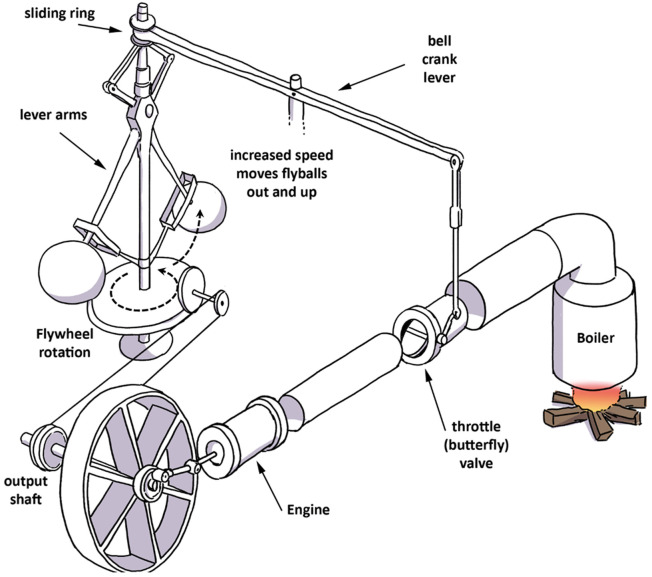
Watt’s centrifugal governor. The output shaft from the locomotive’s rail wheel drives the flywheel of the governor into rotation. As speed picks up, the flyballs move outward and up pulling on the bell crank lever that controls the aperture of the throttle valve. As the valve closes, less steam reaches the engine reducing its speed.

Huygens is also credited with the invention of centrifugal governors, a mechanical device in windmills that regulates the distance and pressure between millstones ( Hills, 1996). In the case of embodied cognition, scholars refer to the phenomenon of coupling as it was observed in an adaptation of the centrifugal governor for a steam engine by James Watt in 1788: the flyball governor ( Van Gelder, 1995). Until this innovation, controlling the speed of a steam engine was problematic. A schematized explanation of the device’s functions is shown in Figure 1: as engine speed increases, the governor rotates at a faster pace and the balls swing out, closing the throttle valve to regulate the rate of steam entering the cylinders, and thus reducing and controlling the speed of the engine. The point of interest here is that the behavior of the valve and flyball governor are constantly coupled; they display completely synchronized motions. They are interlocked in a fully autonomous and auto-controlling feedback mechanism, characteristic of the coupling phenomenon.

Philosopher Tim van Gelder (1995), a proponent of *dynamicism* in cognitive science argues that this device is a more suitable metaphor for modeling human cognition than the classical computer analogy and sense-model-plan-act framework that is used by cognitivists (see also Brooks, 1991). Since its introduction as an analogy to study and explain cognition, debate over the Watt governor has not abated, and the limitations and merits of this analogy are still being discussed ( Baltieri *et al.*, 2020; Bechtel, 1998; Beer, 2000; Beer & Williams, 2015; Chemero, 2009; Eliasmith, 1997; Seth, 2014; Shapiro, 2019).

For the purposes of this article, I will compare this model with the understanding of hearing science, notably the processes underlying the first physiological stages of hearing, which I describe in detail below. However, first it will be fruitful to address the issues that arise when we adopt this device as an analogy for embodied listening: namely the lack of flexibility and absence of adaptability.

These limitations surface when comparing instances of causation and coupling between the device’s parts on the one hand, and between the brain, body, and environment on the other. Shapiro summarizes the way some scholars use the governor as an analogy for the brain-body-environment connection: “The circle of causality present in the governor involved components such as the throttle valve, the flyballs, and the flywheel. The circle of causality from which cognition emerges comprises the brain, the body, and the environment” (2019, p.156). Yet, this parallelism is somewhat imperfect: it assumes that the relationship between the mechanical parts of the governor and the causality that binds them together is comparable to the relationship and the binding between the brain, body, and environment. One problem with this metaphor is that the biological constituents (such as the brain, body, and other biological organisms found in the environment), as well as the constituents of the sub-systems (e.g. the parts of the ear), are not rigidly locked into one another as in the flyball governor. In light of a review of the ear’s functions, the analogy to Watt’s governor begins to break down because in the governor the constituent parts are mechanically locked, a condition that is not comparable to the characteristics of the human ear. On the contrary, the ear is constituted of flexible interdependent parts, as I will show when discussing transmission and adaptive properties. Sound is not transmitted as is but undergoes various transformations from one part of the ear to the next. This is due to the existence of adaptive constraints (the nature of the body) and adaptive demands (imposed by the environment) that call for versatile and flexible accommodative behaviors, a far cry from the rigid nature of the mechanical device. However, as I will demonstrate, coupling needs not be so steely. Pliability can better capture modes of interaction between systems and within physiological sub-systems such as the ear.

The Watt governor bears abstract similarities to embodied listening but should not be taken as exhaustively representing coupling in its multiple forms. Such an oversimplified analogy risks offering a distorted if not amputated understanding of coupling. Thus, we should consider an alternative device.

Staying in the field of engineering, a more compelling model of coupling might be that of a device made of various shafts joined in a flexible manner to account for adaptive interactions between systems and within systems. A device that can serve as an analogy for coupling should allow its constituent parts to interact with some degree of adaptability and autonomy as with elastomer connection hubs found in machines between two shafts and appropriately called *flexible couplings* (made of an elastic material such as rubber). Such couplings serve as both transducer and buffer ^4^.

**Figure 2.**
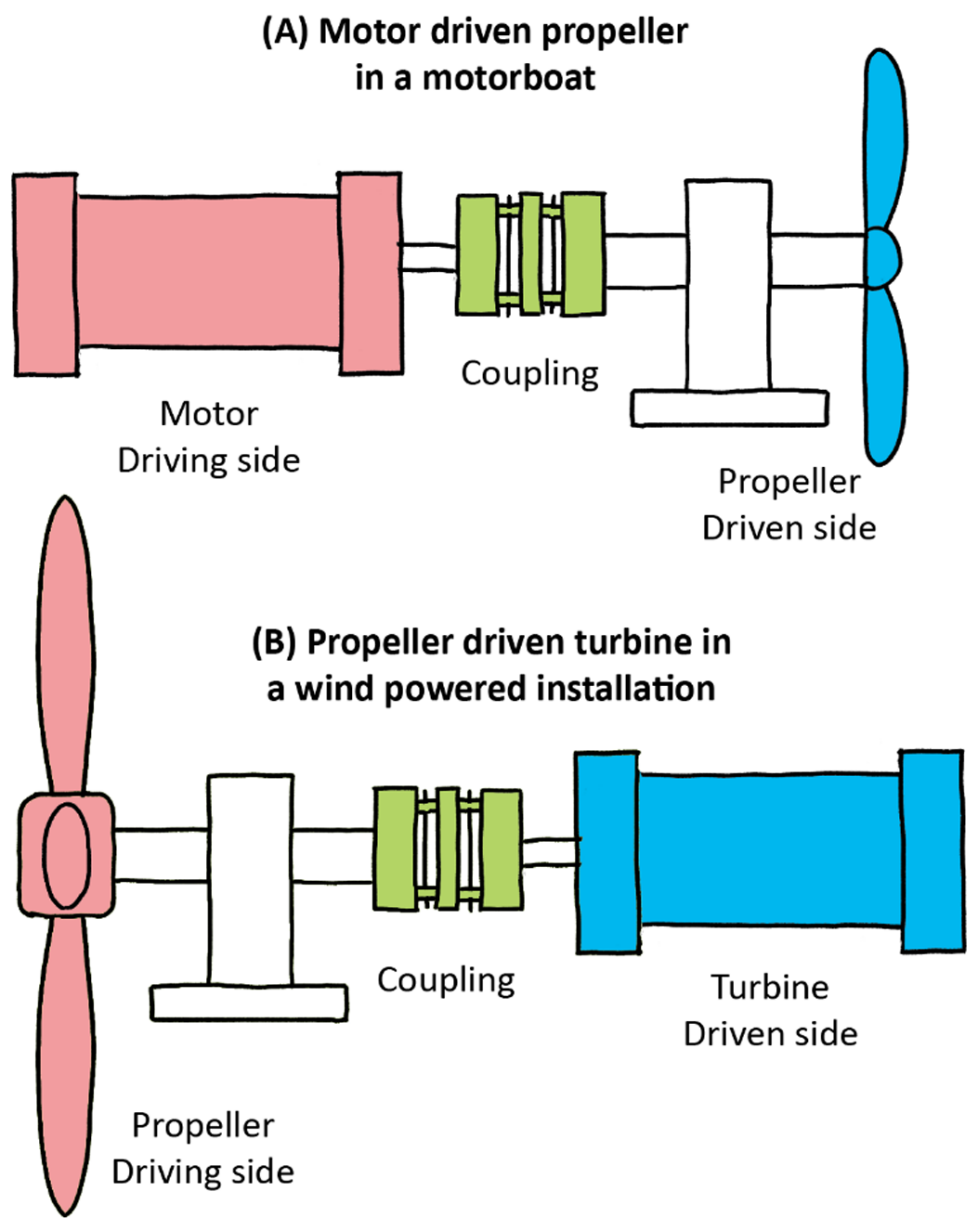
Driving and driven shafts connected by an elastomer coupling device.

A flexible coupling is a mechanical part used to connect two shafts that allows one axis to drive the other with equal torque (spinning velocity); they are thus commonly used in rotary motion applications in machinery (such as vehicles, oil drills, DIY tools, pumps, packaging machinery, rolling mills, etc.) The primary function of these shaft couplings is to transfer power from a driving end to a driven end, such as between a motor and a propeller in an outboard or, conversely, between a propeller and a generator as in a wind turbine ( Figure 2). Moreover, in engineering, coupling devices also serve several other purposes: to connect varying shaft diameters to one another, to accommodate varying degrees of misalignment, to alter the vibration characteristics of rotating parts, to reduce noise, to reduce the transfer of shock loads from shaft to shaft, and to disconnect when overload occurs. The latter characteristics introduce protection, and this is always better achieved by flexible couplings than rigid ones.

Compared with the rigid connections of the flyball governor, the characteristics of flexible interfaces allow for a more faithful illustration of what might be happening between the complex systems of the brain, body, and environment. Within any of those systems, there are coexisting sub-systems. Although all interconnected, different body parts have specific *modus operandi*, with the ear’s main purpose being hearing. The ear is likewise constituted of various “shafts,” transducing acoustic energy from the air to bones, from those bones to liquid, and from those fluids to the nervous system circuity, and then all the way up to the auditory cortex in the brain. The ear is also comprised of built-in devices that protect the driven “shaft” components (i.e. eardrum) from the driving “shaft” components (i.e. ear canal). As with Watt’s governor, there are parallels between the properties of flexible couplings and the basic tenets of embodiment, especially in that coupling precludes the rigid and constrained properties seen in the governor. This model of interconnected shafts enriches the notion of coupling by introducing a less deterministic approach suitable to the adaptive development and behavior of biological systems. Coupling between systems can thus be examined at various levels, as in Table 1.

**Table 1.** Coupling system tiers.

Level	System tier	Example
1	System of systems	coupling between brain-body-world (as in hearing)
2	System	coupling between body parts (as in ear and eyes)
3	Sub-system	coupling within body parts (as in different ear parts)
4	Micro-system	coupling between biochemical elements (as in cochlear hair cells)

Although the Watt governor is used to make a case for coupling at the broad level of a system of systems, coupling characteristics can be found at all levels. This article focuses mainly on the sub-system of the ear to substantiate the design of an embodied listening construct. The main conclusion drawn from the analogy of Watt’s governor with the mind, is that the brain, body, and environment operate in sync, like Huygens’ clocks, and that the mind emerges from those interactions. This ontological view of the mind is quite appealing when making a case for embodiment and situatedness but can also be misleading when we contemplate the incredible complexity of the numerous stages of auditory processing, which are not nearly as straightforward as Watt’s governor. 

From now on, I will consider coupling in relation to the ear in much more detail. Table 2 synthesizes the main analogies that can be drawn between a flexible coupling and the ear.

**Table 2.** Analogies between an elastomer coupling and the ear.

Characteristics of	an Elastomer Coupling	the Ear
Transmission	of torque from a driving end to a driven end	of air vibrations into bone vibrations into liquid vibrations into electrical impulses
Adjustment (general)	by alteration of vibrations	by inhibition or amplification of sound vibrations
Adjustment (dampening noise)	from the engine	by filtering irrelevant sounds and noise (external or internal)
Adjustment (dampening amplitude)	by reducing the transmission of shock loads	by contraction of the stapedius muscle (acoustic reflex) ^5^

Transmission and adjustment account for the processes whereby a device accepts energy from a source in one form and transforms it into a different form as it transfers it to another device. The recipient will act in turn as an emitter and send the energy to another recipient and so on until it reaches its destination (e.g. in the case of the ear, the auditory cortex in the brain is the endpoint). Transmission and adjustment are co-occurring operations. Adjustment comprises amplification (anatomical and physiological) and inhibition or dampening (filtering irrelevant sound or noise whether external or internal).

### Transmission

**Figure 3.**
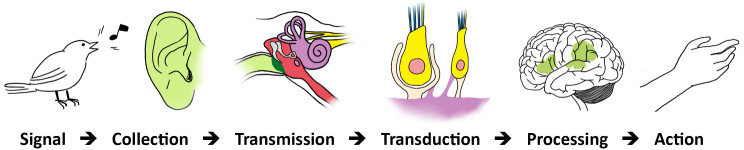
Auditory sensory processing. Signal (acoustic) → Collection: Air vibrations (from open field) → Transmission: Sensory Stimulus (from air to bone to liquid) → Transduction (into electrical impulses) → Processing: Integration (subcortical centers) / Sensation / Perception (auditory cortex) → Action (or reaction).

Instances of coupling can be observed at several stages in the operations constituting sensory processing: during collection, transmission, and transduction ( Figure 3).

To appreciate the subtleties of coupling, a review of the transmitting and adaptive properties of the anatomical ear is warranted ^6^. How does sound travel from the outside open field to the brain? First, I will describe the transmission “shafts” that make up the various anatomical parts of the ear. Then I will review how they interact through adaptive processes (in section *Adjustment* below). Four major transmission processes are explained: A) from air to air; B) from air to bone; C) from bone to liquid; and D) from liquid to electrical signal.

***(A) From air to air (from open field to outer ear)***

**Figure 4.**
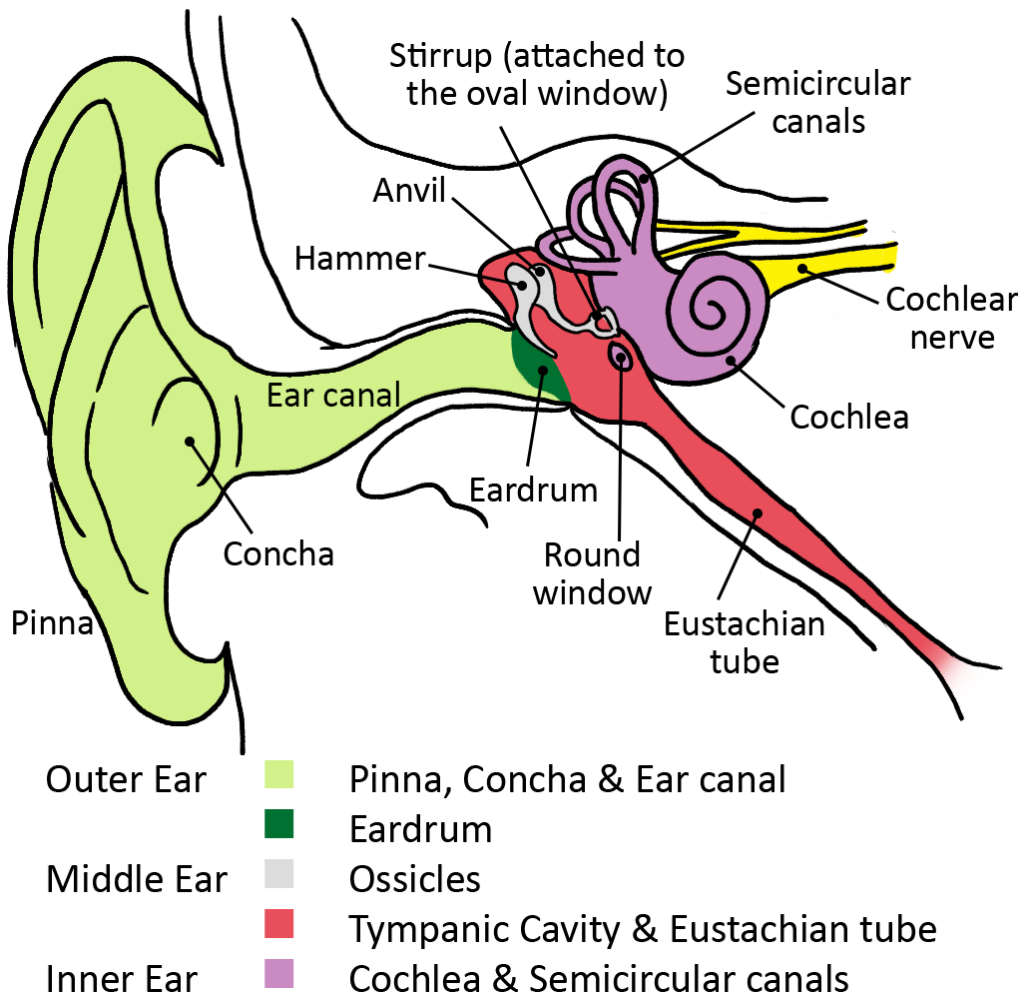
Anatomy of the ear.

The first “hub” and “shaft” are the pinna ^7^, the concha, and ear canal that make up the outer ear (see Figure 4). Sound waves are first funneled by the pinna (or auricle), the outmost visible part of the ear, into and through the auditory canal. The outer ear amplifies and dampens airborne sound energy, leads it to the eardrum, and causes it to vibrate.

***(B) From air to bone (from outer ear to middle ear)***

The eardrum “hub” connects the outer ear “shaft” to the middle ear “shaft,” or ossicular chain, allowing for the transformation of airborne acoustic energy into bone conducted vibrations (in audiology this is referred to as air-conduction and bone-conduction). The eardrum induced vibrations are conveyed behind the eardrum to the middle ear “shaft” consisting of the three smallest bones found in the human body: the hammer, anvil, and stirrup (scientifically labeled as the malleus, incus, and stapes).

***(C) From bone to liquid (from middle ear to inner ear)***

These three ossicles serve as an intermediary bone conduction “shaft” between the outer ear (air conduction) and the inner ear (liquid conduction). They allow for the efficient transfer of energy from one element (air) to another (fluid). The stapes, shaped like a stirrup, pushes into a small oval window that opens onto the cochlea (the snail-like pea seen in Figure 4). The tread of the stapes oscillates against the window displacing the fluids inside of it. 

***(D) From liquid to electrical signals (from inner ear to nervous system)***

Lymph fluids fill the spiraled tubes of the cochlea. When the fluids are moved by the stirrup bone, they cause undulation of the basilar membrane, a thin tissue that divides the cochlear tubes into parallel corridors. According to the frequency of the sound, laminal waves appear on the membrane, which sweep the hairs sitting atop the rows of neurons that line the cochlear tubes. Hair cells then convert these waves (mechanical energy) into electrical pulses (graded receptor potentials), which travel along the auditory nerves so they can be integrated by sub-cortical networks and then processed and perceived by the auditory cortex (for a more detailed account see Musiek & Baran, 2020).

From this brief overview of how acoustical energy is translated into electrical nerve signals, we can see that, although sensory processing happens within milliseconds, it is a tiered process that transforms energy via conductors made of various materials (including air, bone, liquid, and nerves). Thus, coupling in the sense of Watt’s governor is not happening. Rather, the auditory process is the result of a plurality of adapted coupling events.

### Adjustment

Adjustment processes include both amplification properties (exacerbating relevant sound) and inhibitive properties (filtering irrelevant noise whether external or internal) of the outer ear, the eardrum, and the ossicles.

Now, with an idea of how acoustic energy is transported to the nervous system in place, let us examine the adaptive processes that occur along the transducing line of operations. These adaptive processes are hallmarks of embodiment, the notion that the mind forms through repeated interaction with its environment by perceiving it with the senses. Adaptation means that there are constraints and opportunities offered to the nervous system by the very structure of the body that is transducing the information. Because sensory inputs are preprocessed by the body itself ( Chiel & Beer, 1997) the signal that is sent to the nervous system is an adapted version of signals from the environment. In the same way that a rubber coupling can influence how energy is transferred between two shafts because of its inherent physical properties (material, shape, size, thickness, etc.), so do the physical properties of the ear influence the quality and quantity of acoustic information that will be transduced through the anatomical shafts all the way to the brain. A description of the relevant morphology and physiology of the outer, middle, and inner ear structures will help us understand how this occurs.

Before describing these adaptations, notice that the mind also acts upon the body and environment through its unique perceptions and the actions that the mind takes in conjunction with the body in the world, creating a feedback loop that can toggle the perceptual mechanisms (top-down mechanisms). Since this occurs in the central auditory system (the nervous system), it is beyond the scope of the present paper ^8^. A look at adjustments made by the peripheral auditory system (the anatomical ear) will suffice to demonstrate that listening as a cognitive construct is pre-processed by the body and should therefore be regarded as embodied. I will now explain the adaptive properties shared by the different “shafts” and “hubs” of the peripheral auditory system, serving as either amplifiers or filters or both when transporting vibrations to the central auditory cortex.

***(A) The pinna: its shape, size, and angle affect collection of sound and thus its perception***

Obviously, animal ears come in a wide variety of shapes and sizes. Take the pointed ears of fennec foxes, the great flaps of an elephant’s ears, or the ears of a mole, just tiny holes hidden under its fur. Different ears serve different functions among species but most capture different swathes of sound, accounting in part for the variability in hearing ranges. Less obvious but equally significant, varying shapes among human ears likewise capture varying degrees of acoustic information ( Shaw, 1974).

The first “hub” serving as an interface between the open field sound environment and the “shaft” or conduit to carry airborne signals to the eardrum is the outer, C-shaped flap of the ear, called the pinna. The very shape of the human ear has consequential effects on how we hear ( Spagnol *et al.*, 2012).

When sound reaches the outer ear, not all sounds are gathered equally and sent through the ear canal. Because of the intricate ridges and depressions on the surface of the pinna, frequencies are not evenly amplified and filtered. In fact, the pinna acts as an amplifier for mid-range frequencies, as a filter at low frequencies, and as a direction-dependent filter for higher frequencies in order to enhance spatial perception ( Musiek & Baran, 2020, p. 51).

Wavelengths of frequencies the size of or smaller than the pinna will be more readily funneled into the ear canal. In addition, the swirly shape of the pinna acts as a resonator to enhance mid-range frequencies between 2000 Hz and 7000 Hz ( Ballachanda, 1997), the range typical of human voices. These folds and recesses enhance sounds that are meaningful to humans, leaving other pitches untouched or reducing background noise usually found in lower frequencies.

Indeed, lower frequencies with wavelengths larger than the pinna more readily spill around the ear. The size of the auricle is directly correlated with the size of the frequencies that can be captured. Take an elephant: their ears can detect frequencies lower than any known to land mammals ( O’Connell-Rodwell, 2007). With their large, fanned-out, and movable pinna, they can pick up infrasound not detectable by the human ear. Variations between species are salient and well known; similarly, anatomical differences in the outer ear alter the external ear transfer function from one person to the next ( Shaw, 1974). Big ears, small ears, flat, flabby, or more cartilaginous ears already account for some of the variability in individual sound perception.

Because the pinna is at an angle turned slightly to the front, the ear gathers more information from the soundscape a person is facing rather than from what is behind them. As humans usually face each other when speaking this is a fitting adaptation. The ear flaps have the effect of somewhat dampening how much background noise and other sounds arriving from behind are able to reach the ear canal, a phenomenon known as the *pinna shadow effect*. This discrepancy in humans’ capacity to pick up sounds behind and in front of them also allows for enhanced detection of sound sources. This is why people with behind-the-ear (BTE) hearing aids have their ability to determine where a sound is coming from negatively impacted. In this instance, the pinna shadow effect is impaired, and the hearing-aid user is, therefore, less able to distinguish between front and back ( Van den Bogaert *et al.*, 2011). People with flatter ears could also conceivably experience difficulties with sound location, even though they are able to pick up sounds from behind better. On the other hand, cupping the hand behind the ear to concentrate sounds coming from a source in front, boosts mid to high frequencies by as much as 8 dB — a 250% amplitude — and attenuates similar sound spectrums originating from the back by up to 9.5 dB — about 300% ( Barr-Hamilton, 1983). These variations highlight the difference it might make to have flattened cauliflower ears or to have protruding ones like Will Smith’s. In short, the shape, size, and angle of the pinna affect the collection of sound into the ear canal and thus influence perception, that is to say, the way one experiences surrounding soundscapes.

The overall *pinna effect* (sound filtering and localization) is so profound on hearing, that some hearing aid manufacturers are now moving away from BTE devices to engineering receivers placed directly inside the ear canal, making it capable of utilizing the pinna effect unique to each user. They know how influential the body is on perception and thus cognition. Indeed, each individual brain has been perceiving sound shaped by the person’s unique pinna since the day they were born, building mental models of the ambient soundscapes. So, when receivers are placed behind the ear flange, most of the neuro-acoustic benefits developed by the brain from years of experience with sound is lost. Therefore, as Groth *et al.* remark, in-canal hearing aids allows the auditory system to “organically select, separate, and integrate sonic features delivered via our ears” ( Groth *et al.*, 2020; p.17). Instead of “reconstructing” sound from behind the ear, this ecological perspective underscores the advantages of using the existing embodied listening profile of the user to enhance smoother sound processing and intelligibility

***(B) The ear canal: its shape (twists and turns), size (diameter and length), and material affect transmission of sound and thus perception.***

Behind the pinna that acts as a “hub,” lies the first anatomical “shaft,” the external auditory meatus, more commonly known as the ear canal. Because of its cylindrical shape, it acts as a resonator, altering the acoustic signal that it carries to the eardrum. This membrane is compliant to the incoming waves, but, because it closes the end of the tube it also reflects a minute part of the energy back into the conduit, accounting for the resonance in the ear canal. It is a bit like blowing into the top of a glass bottle: because it is closed at its base the bottle will resonate at a particular pitch according to its shape. Likewise, the ear canal resonates in a particular way because of its size and shape. It acts as a quarter-wave resonator, that is, it boosts wavelengths that are 4 times longer than its own length. Because the average ear canal length in adults is approximately 2.5 to 3 cm long, it will enhance wavelengths of 10 to 12 cm. These wavelengths fall into the 3000 to 4000 Hz range ( Dallos, 1973). Unsurprisingly, the human voice is mostly distributed within that bandwidth.

As a consequence of the morphology of the outer ear, which is comprised of the pinna, concha and ear canal, the spectrum of sound delivered to the eardrum emphasizes frequencies useful for vocal communication in humans. Conversely, lower and higher frequency ranges are de-emphasized. This means that “the signal that reaches the eardrum is not the same signal that is being delivered by the sound source” ( Musiek & Baran, 2020, p. 55). The outer ear is partial to what is deemed as the most important sound to humans, that is, the voices of their fellow human beings

***(C) The eardrum (or tympanic membrane): its shape, size, angle, stiffness, and thickness affect transduction of sound and thus perception.***

The second “hub” serving as an interface between the ear canal “shaft” (outer ear) and the ossicular chain “shaft” (middle ear) is the eardrum. This “hub” serves as a transducer, converting sound energy arriving through the ear canal into mechanical energy by way of the handle of the hammer bone (malleus), which is attached against the back of the eardrum.

Although often compared to the taut surface of a drum, the eardrum is not flat but cone-shaped, with the rim facing the ear canal and the tip of the cone (umbo) protruding about 2mm back into the middle ear cavity ( Alvord & Farmer, 1997). It is not a coincidence that cone-shaped membranes are also used in home stereo equipment and speaker system diaphragms. Woofers have large flexible membranes for low frequencies, squawkers have medium-sized membranes for midrange frequencies, and tweeters have small stiff membranes for high frequencies. Technically, this makes sense, as separating membranes into three types enables better concentration of sound direction and power for each bandwidth. By contrast, humans have only one membrane, the size and tension of which does not allow for it to vibrate at the full spectrum of arriving frequencies. Since the membrane cannot operate large displacements, it does not vibrate to low-frequency sounds very well (as does a woofer, with its large supple skin to allow it to generate low sounds). The eardrum’s mass inertia also limits the transfer of high-frequencies (which the small stiff membrane of the tweeter does well). Thus, energy transfer in the eardrum is most efficient in the range of 800 to 6000Hz ( Emanuel & Letowski, 2009, p. 162). In short, the human ear works best as a squawker.

Furthermore, the dimensions and thickness (number of layers) of the membrane show considerable individual variation as well as dependence on age and sex ( Graham *et al.*, 1978). Hearing scientists agree that there is no such thing as an “average human eardrum” ( Van der Jeught *et al.*, 2013). In other words, membrane stiffness differs among individuals, resulting in a high degree of variability when sound energy is converted into mechanical energy through the three small bones in the ear.

Any changes in the dimensions of the pinna, concha, ear canal, or eardrum structures will consequently alter the characteristics of received sound before they reach the brain. Variation is usually inherited, but distortions can be incurred by, for example, physical injuries, occlusion (ear mold), or ageing — conditions that will affect what a person hears, what they become used to hearing, and, ultimately, their overall perception of their acoustic surroundings.

**Figure 5.**
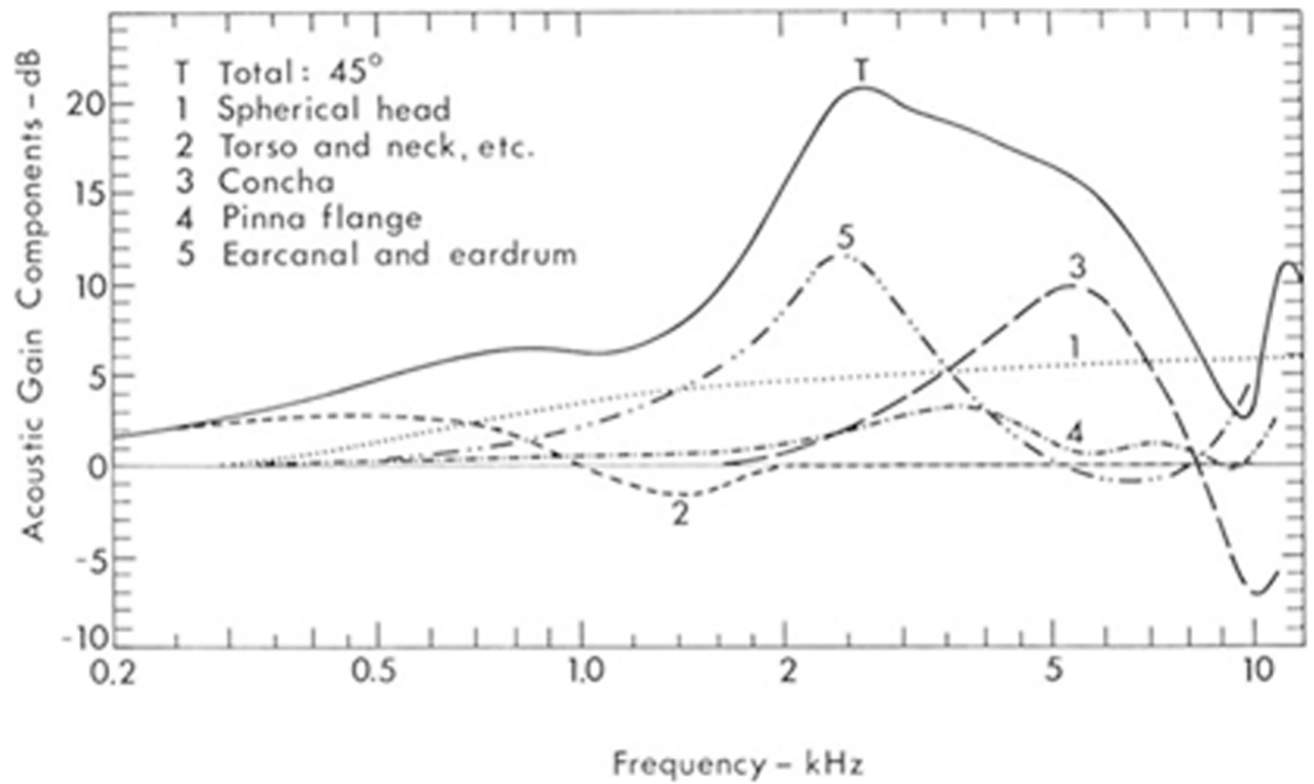
Intensity gain in the external ear. (From Shaw, 1974, with permission from Springer Science and Business Media.)

To put all these factors into perspective, we can refer to Shaw’s (1974) compilation of data on the combined effects of sound amplification and dampening by the outer ear, as seen in Figure 5. Shaw demonstrates that the head and body also play a non-negligible role in sound diffraction and reflection, which can be seen in curves 1 and 2. As discussed above, the pinna has a strong intensifying role but mostly at the level of the concha (the doorway to the ear canal, seen in curve 3). The helix and antihelix curvatures of the pinna have a lesser distorting effect (curve 4). But none of these structures have as prominent an effect as the ear canal and eardrum (curve 5). The greatest average pressure gain as a result of all these combined effects is between 2000 and 3000 Hz (curve T), the bandwidth humans use most for speech intelligibility. The ear resonance is improved at its peak at 17 dB around 2700 Hz, an amplification of about 700%.

Although the workings of the external ear account for a great deal of variability in what humans capture from their acoustic environment and for the particular emphasis given to speech sounds, this is but the first step in sound transmission. Let us look now at what happens in the middle ear.

***(D) The ossicular chain: its shape, size, angles, and flexibility affect transduction of sound and thus its perception***

The middle ear “shaft” looks like a lever made of the three bones commonly known as the hammer, anvil, and stirrup. This bony “shaft” cumulates functions of conduction, protection, transduction, and amplification.

Its main role is to ensure that air-conducted vibrations from the outer ear are properly transferred and transformed into liquid-conducted vibrations in the inner ear by means of mechanical energy. The hammer handle set in the back of the eardrum receives sound-induced vibrations from the tympanic membrane, and, in conjunction with the anvil, causes the stirrup’s footplate to oscillate against the oval window of the cochlea, where encased fluids are stirred. Because sound, as it exists in the air, does not naturally penetrate fluids, the ossicular chain acts as a coupling device between air and liquids. As an analogy, take the experience of going underwater in a swimming pool: all surrounding sounds are suddenly dampened. This is because airborne sounds bounce off the surface of the water. For sound to effectively penetrate an aqueous environment, an adaptation is necessary. The ossicular chain provides this conversion by means of the three mechanisms known in audiology as “ossicular coupling.”

**Figure 6.**
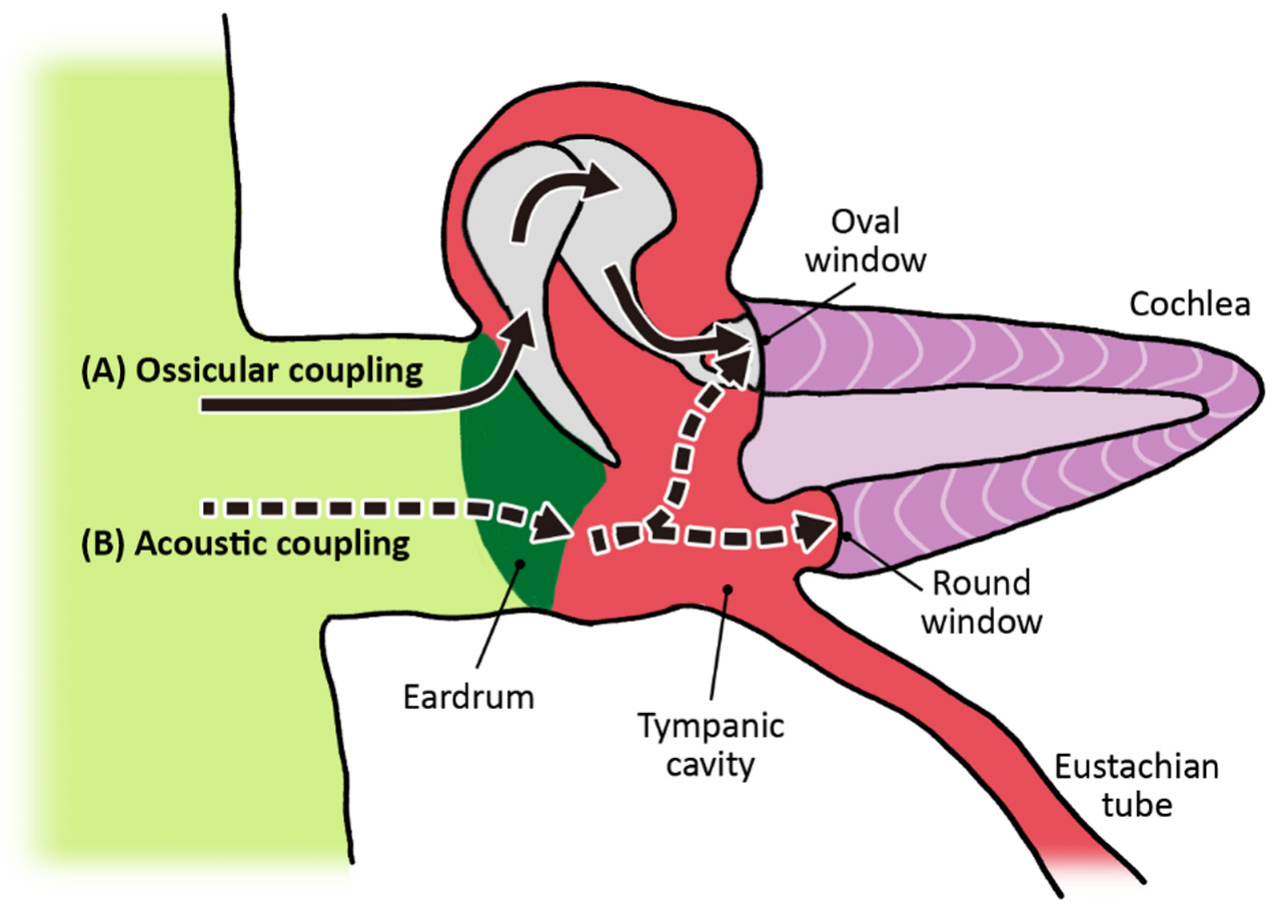
Ossicular and acoustic coupling. ( **A**) Most sound vibrations are transduced through the ossicles to the oval window. ( **B**) Residual sound is transferred directly by the eardrum into the tympanic cavity and slightly affects both windows. The spiraled cochlea ducts are elongated and modified in this view.

Ossicular coupling occurs through three simultaneous mechanisms: i) area difference ratio (eardrum to stirrup’s footplate); ii) lever action (hammer to anvil); and iii) curved membrane effect (eardrum to hammer) ( Figure 6). I explain each of these mechanisms below.

*(i) Area difference ratio (eardrum to stirrup’s footplate)*

Because the area of the eardrum (45mm²) is much larger than the tread of the stirrup (3.2mm²), sound vibration that strikes the eardrum is pressed down into the much smaller surface of the footplate, increasing pressure while reducing speed and displacement, and thereby transforming the mechanical energy into hydraulic energy. Since the surface of the eardrum is around 17 times larger than the oval window, the sound pressure is condensed, leading to an amplification of about 25 dB.

To relate to how this works, Emanuel & Letowski (2009, p.161) analogize this mechanism to a pair of snowshoes: because they are large and flat, a person’s body weight can be dispersed over the wide surface of the snowshoes, allowing a person to walk over unpacked snow. If a person puts on regular boots, which have soles with a smaller surface area, then the same body weight will likely push them through the loose snow. The stapes is like a pair of regular boots: it pushes in and out of the oval window, pressurizing the liquid behind the window into hydraulic action.

*(ii) Lever action (hammer to anvil)*

**Figure 7.**
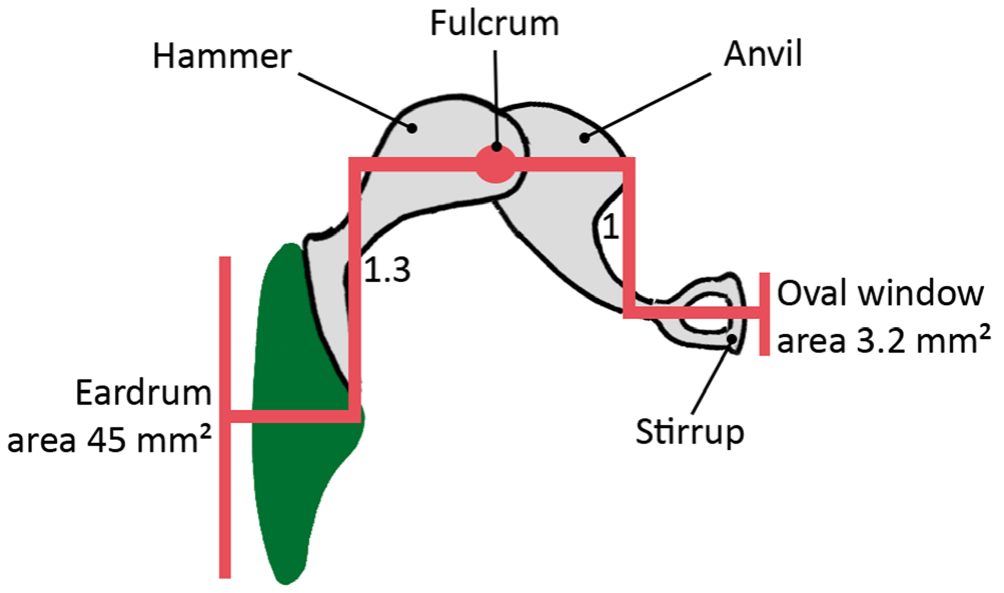
Ossicular coupling. Area difference between the surface of the eardrum and the stirrup plate fitted onto the oval window of the cochlea. Lever action between the hammer and the anvil.

**Figure 8.**
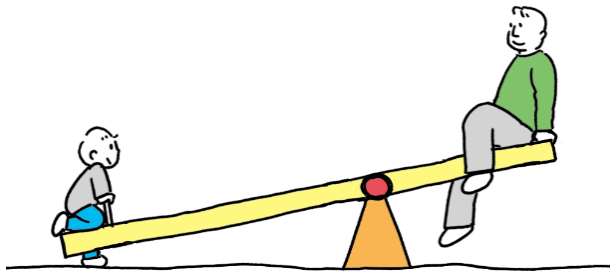
Ossicular coupling. First-class lever.

As seen in Figure 7, the handle of the hammer is 1.3 times as long as the anvil. This mechanism produces an action that converts pressure from the hammer into higher pressure by way of a short lever action at the tip of the longest side of the anvil. To understand how this works, we can draw an analogy to a playground seesaw ( Figure 8): if we were to slide the plank over the fulcrum so that one end of the plank was longer than the other, it would make it easier for a child to lift the weight of an adult. Similarly, as a result of the unequal lengths of the bones, the hammer is able to hoist the anvil with greater ease and power.

*(iii) Curved membrane effect (eardrum to hammer)*

**Figure 9.**
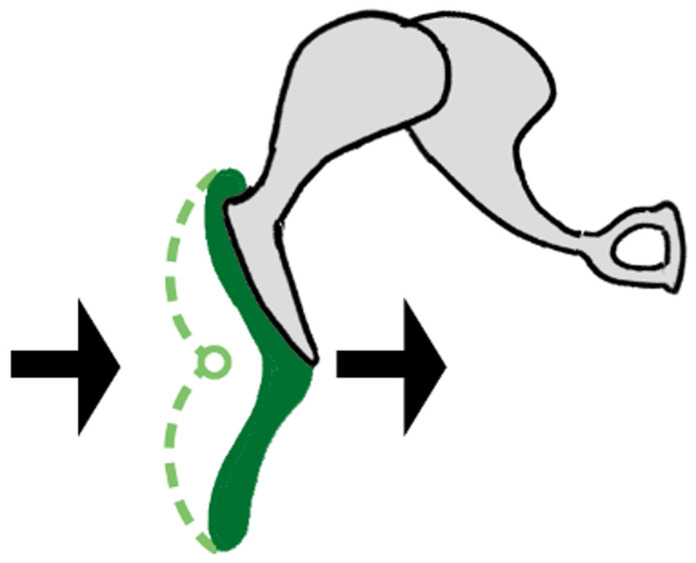
Ossicular coupling. Buckling of the eardrum.

The third phenomenon in ossicular coupling is a result of the eardrum’s position in the middle ear cavity. The Eustachian tube (see Figure 4) equilibrates the air pressure in the middle ear with that of the external atmospheric pressure, permitting the eardrum’s membrane to sit in its most natural and neutral position. But, as air pressure hits the eardrum from the ear canal, it creates a depression between both sides of the diaphragm, and, as a result, the eardrum makes a buckling motion, which increases the force fourfold, focusing the sound waves onto the hammer bone ( Figure 9).

The coupled motion of the tympanic membrane and ossicles, including the stapes footplate, generate a combined magnification in pressure of about 45 times (an increase of 30 to 35 dB). This mechanical thrust delivers enough power to efficiently transfer air-borne sound vibrations into fluid-borne vibrations. Ossicular coupling provides us with an exemplar of how coupling in the ear is adaptive, constraining certain sounds and boosting others. Moreover, it highlights the variegated features of coupling: overall, the coupling of open-field sound with a sound-image in the cortex is the result of multiple cascading instances of coupling, each bearing distinctive modes of operation as illustrated in the examples above.

Incidental to ossicular coupling, there is a second pathway by which the middle ear transmits energy to the cochlea called *acoustic coupling*. Some of the energy that hits the lower part of the tympanic membrane is not transferred to the ossicles but reverberates directly into the air-filled cavity of the middle ear (where the Eustachian tube connects). This has only a slight effect on the two windows placed at each end of the perilymph filled corridors of the cochlea (see Figure 6). Yet, Peake and colleagues have shown that “a loss in ossicular coupling is consistent with the cochlea responding only to the pressure difference at its oval and round windows (i.e. acoustic coupling)” ( Peake *et al.*, 1992, p.17), and this might be one of the only ways for some people to still capture sound cues instead of becoming completely deaf when ossicular coupling fails.

And, indeed, the efficiency of this ossicular mechanism can be compromised when the ossicles are misshapen or displaced (or nonexistent), either by birth defects or even minor physical traumas, such as a Q-tip injury. Most often injury occurs when the middle ear is deeply affected by repeated otitis media with effusion, especially in children under 2 years old. The lasting negative consequences of such illness on cognition and academic performance have been amply confirmed ( Williams & Jacobs, 2009).

That being said, even among individuals with “normal” ears (no pathology nor malformations) we can detect significant differences of up to -25 dB (a 6 to 7-fold variance in volume intensity) in the hearing response curves. Dallos (1973) noticed that the shape of auditory threshold curves (the string of points at which given sounds are perceptible) is contingent on the sound transmission performances of the outer and middle ear. Goode ( Goode, 1997, p17) suggested that even slight variances, both in the stiffness of the eardrum ( Zwislocki, 1975) and the lever ratio contributing to the force of amplification in the ossicles ( Brenkman *et al.*, 1987), account for the large perceptual differences noted between individuals, actually calling sensitive ears in the top 10% “golden ears” and in the bottom 10% “tin ears” (in Emanuel & Letowski, 2009, p.162).

## Discussion

If it is possible to represent coupling between the brain, body, and environment through the Watt governor, the same model should also be an edifying way to describe coupling in each of the lower-tier systems, such as the body itself (system) or the ear (sub-system). This is precisely what dynamic system theory endeavors to achieve: to provide a model that can describe any dynamic system, from the macro to the micro-level. However, from the above examination of sound transmission processes in the sub-system of the ear, we can see that coupling at this level is not as rigid as the Watt governor model might suggest. Coupling is not a straightforward process but involves various alignments, where some information is attenuated and other information is amplified. A more appropriate analogy to describe coupling for a sub-system, such as the ear, might be the elastomer coupling devices found in between shafts in machinery, which are similar to the buffer and amplification properties of the ear. Yet, when equating the Watt governor mechanism or the flexible shaft-to-shaft mechanism to the brain-body-environment system, a literal-minded thinker might take it as over-constrictive, but a more figurative-minded reader will see its value in the way it illustrates a concept. The above inquiry into the multifaceted nature of coupling exhibited by the ear makes for a more nuanced understanding of the very concept of coupling. By itself, the analogy of the governor does not render the diversity of coupling features to be found in the ear. However, the combination of various analogies for differing instances of coupling, such as the flexible coupling hubs, shafts, musical instruments, audio-speakers, snowshoes, the seesaw, and other metaphors paint a more vivid and variegated picture on the canvas of dynamic systems theory.

At this point, one might object that coupling, outfitted in such motley apparel, has nothing to do with dynamic systems theory since it does not fit with the idea of non-linearity so typical of systems displaying dynamic properties. So far, coupling in the ear has indeed been depicted as a linear shaft-to-shaft operation, arrayed in manifold coupling mechanisms. However, this approach does not in any way rule out the co-occurrence of non-linear phenomena. Both linear and non-linear approaches are complementary to shedding light on the complexity of coupling in a dynamic system. Coming back (one last time) to the Watt governor and the engine it is part of, we can observe both linear and non-linear phenomena. In the following description, note how *which* is used to translate linear causality from one mechanical part to another (an asynchronous action) and *as* is used to convey the idea of non-linear causality as in coupling (a synchronous action): shoveling coal in the boiler increases the steam load, *which* speeds up the engine, *which* entrains the horizontal flywheel *as* it drives the central shaft of the governor to rotate faster, *as* it propels the flyballs on the arms to move outward and upwards, *as* it causes the arms to pull down on the sliding ring, *as* it moves the bell crank lever, *as* it reduces the opening of the butterfly valve *which* decreases the amount of steam *which* lowers the speed of the engine. The overall operations are generated through mechanisms displaying both linear and non-linear traits.

What about the ear? Research in hearing science has paved the way to provide non-linear models of the acoustic and structural coupled systems of the ear ( Ihrle *et al.*, 2013). Briefly put, these scientists explain how sound transfer is affected as the resting position of the middle ear architecture alters when receiving sound. The eardrum is affected by the elasticity of both the ear canal in front of it and the tympanic cavity behind it: both chambers are dynamical elastic bodies, not static entities as described in anatomical books. This means that each structure influences others, a distinctive property characteristic of a dynamic system. This study shows that, for example, the pressure differences encased in the cavities on both sides of the eardrum affect its position, while the complex geometry of the eardrum itself further influences the motions of the ossicular chain. The overall effect is additional modulation of how sound is transferred through the middle ear. From the point of view of physics, the transfer of acoustic energy is not just deterministic (linear) but also probabilistic (non-linear).

Having observed this, we can turn back to the main discussion, which aims at explaining how coupling, from both a linear and non-linear perspective, helps us see that sound perceived by the brain after its transfer through various internal channels does not exactly correspond to the sound emitted from a given source. This supports the claim that the building blocks of the mind are influenced by the very nature of the body (adaptive constraint), from which acoustical input (adaptive demand) — or for that matter any sensory input — is collected and transferred to higher levels of integration in the brain.

Furthermore, coupling effects that are important to human variability can also be found between the cochlea and the auditory nerves that send signals to the brain. This will be explored in a sequel article, when describing self-organizing systems. For the time being, what emerges from a close inspection of the coupling functions in the ear, is that given the premise that the mind’s development is contingent on perception, which itself varies according to physical properties, the mind’s configuration must at least be partially reliant on the body’s anatomy and physiology, thus making a case for embodied listening and, by extension, embodied cognition.

As we have seen, specific anthropometric features of people, i.e. size, shape, position (angle) of any of the anatomical parts of the outer, middle, and inner ear ^9^, vary widely depending on age, gender, and genetic factors and have cascading influences on cognitive performance. Moreover, audiologists have concluded that the specific resonance characteristics of these parts vary greatly as a result of these factors. Because auditory thresholds are different in individuals (i.e. the sounds a person can perceive at a given volume), this can do a lot to explain the varying responses each person will provide to incoming auditory information. Indeed, the way a person experiences the soundscape around them may be said to be unique. We usually take for granted that nearly everyone hears the same way (save for our elders and others with hearing loss). However, we often fail to realize to what extent there exists considerable variance in listening ability among the people we interact with on an everyday basis. Whether a teacher is faced with a class full of kids, teenagers, or adults, they would do well to realizing that no two sets of “ears” are the same and to empathize with the fact that each individual has a different perceptual system, which impacts the formation and the workings of their mind. Academic achievement can be affected positively, merely by virtue of possessing “golden” ears, or negatively, through no fault of one’s own, as a consequence of having “tin ears.” If, from the outset, individual differences prevail at the physical level, thereby impacting academic outcomes, the role of educators in trying to mitigate these inequalities and provide for more equitable learning opportunities becomes even more important.

In closing, we should consider the following conclusions:

1. *Coupling in hearing is variegated:* both linear and non-linear coupling phenomena make allowances for an overall description of how sound is processed by the outer and middle ear;
2. *Hearing is a pre-perceptual mechanism:* it impacts listening through the adaptive constraints imposed by the ear’s anatomy and physiology;
3. *Anatomical variability in individuals is the norm:* by extension, what a person perceives and how they respond, for example in terms of academic achievement, can be surmised as at least partially resultant of such structural singularities;
4. *Listening is embodied:* the structure of the ear creates constraints and opportunities for the emergence of the mind.

Finally, the above review of the external and middle ear mechanisms sheds light on the fact that a constrained definition of coupling alone, as previously discussed, cannot account for the complexity of auditory processing. And we have only glimpsed the tip of the iceberg. In sequel articles, we will delve into the waters of auditory integration and explore how it gives rise to further sophistication, which both substantiates dynamic systems theory as a means of understanding the emergence of an embodied mind and adds nuance to this framework. I will also demonstrate how further embodiment features, as well as environmental factors, help explain large variations in listening from one individual to the other. These insights should be of use to educators, who are trying to understand what they can do to provide their learners with better listening strategies and opportunities. This will be made possible by incorporating the body and environment into the general picture of an embodied and situated listening paradigm, a project that is under development.

## Data availability

No data are associated with this article.

## References

[ref-1] Alvord LS Farmer BL : Anatomy and orientation of the human external ear. *J Am Acad Audiol.* 1997;8(6):383–390. 9433684

[ref-2] Ashcraft N Tran A : Teaching Listening: Voices from the Field.Teachers of English to Speakers of Other Languages, Inc. 1925 Ballenger Avenue Suite 550, Alexandria, VA 22314,2010;225. Reference Source

[ref-3] Aziz-Zadeh L Wilson SM Rizzolatti G : Congruent embodied representations for visually presented actions and linguistic phrases describing actions. *Curr Biol.* 2006;16(18):1818–1823. 10.1016/j.cub.2006.07.060 16979559

[ref-4] Bailey KM : Teaching Listening and Speaking in Second and Foreign Language Contexts. Bloomsbury Academic.2020. Reference Source

[ref-5] Ballachanda BB : Theoretical and applied external ear acoustics. *J Am Acad Audiol.* 1997;8(6):411–420. 9433687

[ref-6] Baltieri M Buckley CL Bruineberg J : Predictions in the eye of the beholder: an active inference account of Watt governors.In *Artificial Life Conference Proceedings*. One Rogers Street, Cambridge, MA 02142-1209 USA journals-info@ mit.edu: MIT Press.2020;121–129. 10.1162/isal_a_00288

[ref-7] Barr-Hamilton RM : The cupped hand as an aid to hearing. *Br J Audiol.* 1983;17(1):27–30. 10.3109/03005368309081479 6860820

[ref-8] Barsalou LW : Grounded Cognition. *Annu Rev Psychol.* 2008;59:617–645. 10.1146/annurev.psych.59.103006.093639 17705682

[ref-9] Beer RD : Dynamical approaches to cognitive science. *Trends Cogn Sci.* 2000;4(3):91–99. 10.1016/s1364-6613(99)01440-0 10689343

[ref-10] Beer RD Williams PL : Information processing and dynamics in minimally cognitive agents. *Cogn Sci.* 2015;39(1):1–38. 10.1111/cogs.12142 25039535

[ref-11] Bechtel W : Representations and cognitive explanations: Assessing the dynamicist’s challenge in cognitive science. *Cogn Sci.* 1998;22(3):295–317. 10.1207/s15516709cog2203_2

[ref-12] Bodie GD Wolvin AD : The Psychobiology of Listening: Why Listening Is More Than Meets the Ear. *The Oxford Handbook of the Physiology of Interpersonal Communication*.2020;288. 10.1093/oxfordhb/9780190679446.013.16

[ref-13] Braga J Samir C Risser L : Cochlear shape reveals that the human organ of hearing is sex-typed from birth. *Sci Rep.* 2019;9(1):10889. 10.1038/s41598-019-47433-9 31350421 PMC6659711

[ref-14] Brenkman CJ Grote JJ Rutten WLC : Acoustic transfer characteristics in human middle ears studied by a SQUID magnetometer method. *J Acoust Soc Am.* 1987;82(5):1646–1654. 10.1121/1.395156 3693706

[ref-15] Brown S Brown SR : Listening myths: Applying second language research to classroom teaching.University of Michigan Press.2011;15(1). Reference Source

[ref-16] Brooks RA : Intelligence without reason. *Artificial intelligence: critical concepts.* 1991;3:107–63.

[ref-17] Buck G : Assessing listening.Cambridge University Press.2001. 10.1017/CBO9780511732959

[ref-18] Buck J Buck E : Biology of synchronous flashing of fireflies. *Nature.* 1966;211(5049):562–564. 10.1038/211562a0

[ref-19] Cauldwell R : Phonology for Listening: Teaching the Stream of Speech.Birmingham, England: Speech in Action.2013. Reference Source

[ref-20] Cauldwell R : A Syllabus for Listening: Decoding.Birmingham, England: Speech in Action.2018. Reference Source

[ref-21] Chemero T : Radical Embodied Cognitive Science.Cambridge, MA: MIT Press.2009. Reference Source

[ref-22] Chiel HJ Beer RD : The brain has a body: Adaptive behavior emerges from interactions of nervous system, body and environment. *Trends Neurosci.* 1997;20(12):553–557. 10.1016/s0166-2236(97)01149-1 9416664

[ref-23] Conti G Smith S : Breaking the Sound Barrier: Teaching Language Learners How to Listen.Independently Published.2019. Reference Source

[ref-24] Cutler A : Native listening: Language experience and the recognition of spoken words.MIT Press.2012. Reference Source

[ref-25] Dallos P : The auditory periphery biophysics and physiology.Elsevier.1973. Reference Source

[ref-26] De Vega M Glenberg A Graesser A : Symbols and embodiment: Debates on meaning and cognition.Oxford University Press.2012. 10.1093/acprof:oso/9780199217274.001.0001

[ref-27] Descartes R : Discours de la Méthode Pour bien conduire sa raison, et chercher la vérité dans les sciences. [Discourse on the Method of Rightly Conducting One's Reason and of Seeking Truth in the Sciences.]A Leyde, De l'imprimerie de I. Maire.1637. Reference Source

[ref-28] Emanuel DC Letowski T : Hearing science.Wolters Kluwer Health/Lippincott Williams and Wilkins.2009. Reference Source

[ref-29] Eliasmith C : Computation and dynamical models of mind. *Minds Mach.* 1997;7(4):531–541. Reference Source

[ref-30] Field J : Rethinking the second language listening test: from theory to practice.Equinox Publishing.2019a. Reference Source

[ref-31] Field J : Second language listening: current ideas, current issues.In Schwieter JW, Benati A (ed(s).). *The Cambridge Handbook of Language Learning.*Cambridge: Cambridge University Press,2019b;283–319. 10.1017/9781108333603.013

[ref-32] Field J : Listening in the language classroom. *ELT journal.* 2010;64(3):331–333. 10.1093/elt/ccq026

[ref-33] Flowerdew J Miller L : Second language listening: Theory and practice.Ernst Klett Sprachen.2005. 10.1017/CBO9780511667244

[ref-34] Flowerdew J Long MH Richards JC : Academic listening: Research perspectives.Cambridge University Press.1994. Reference Source

[ref-35] Geffner D Ross-Swain D : Auditory processing disorders: Assessment, management, and treatment.Plural Publishing.2018. Reference Source

[ref-36] Goh CC : Second language listening comprehension: Process and pedagogy. *Teaching English as a second or foreign language.* 2014;4:72–89. Reference Source

[ref-90] Goode RL : The ideal middle ear prosthesis. *Middle Ear Mechanics in Research and Otosurgery.* Dresden, Germany,1997;169–74.

[ref-37] Gordon DM : Ant encounters: Interaction networks and colony behavior.Princeton: Princeton University Press.2010. Reference Source

[ref-38] Graham MD Reams C Perkins R : Human tympanic membrane--malleus attachment. Preliminary study. *Ann Otol Rhinol Laryngol.* 1978;87(3 Pt 1):426–431. 10.1177/000348947808700326 655586

[ref-39] Groth J Ruggles D Ellison J : Sizing Up Hearing Aids in the 21st Century: Is There Still Room for Improvement?Retrieved January 6, 2021, 2020. Reference Source

[ref-40] Harnad S : The symbol grounding problem. *Physica D.* 1990;42(1–3):335–346. 10.1016/0167-2789(90)90087-6

[ref-41] Hills RL : Power from wind: a history of windmill technology.Cambridge University Press.1996. Reference Source

[ref-42] Huygens C : Horologium Oscillatorium Sive de Motu Pendulorum ad Horologia Aptato Demonstrationes Geometricae.Paris, France: Apud F. Muguet. (1673, 1966). Reference Source

[ref-43] Ihrle S Lauxmann M Eiber A : Nonlinear modelling of the middle ear as an elastic multibody system — Applying model order reduction to acousto-structural coupled systems. *J Comput Appl Math.* 2013;246:18–26. 10.1016/j.cam.2012.07.010

[ref-44] Lakoff G Johnson M Sowa JF : Review of Philosophy in the Flesh: The embodied mind and its challenge to Western thought. *Comput Linguist.* 1999;25(4). Reference Source

[ref-45] Lynch T : Teaching second language listening.New York, NY: Oxford University Press.2009. Reference Source

[ref-46] Musiek FE Baran JA : The auditory system: Anatomy, physiology, and clinical correlates.Plural Publishing.2020. Reference Source

[ref-47] Nation ISP Newton J : Teaching ESL/EFL Listening and Speaking.Routledge; 2nd Edition.2020. Reference Source

[ref-48] Nemtchinova E : Teaching Listening, Revised.TESOL International Association.2020. Reference Source

[ref-49] Ockey GJ Wagner E : Assessing L2 listening: Moving towards authenticity.John Benjamins Publishing Company.2018;50. 10.1075/lllt.50

[ref-50] O’Connell-Rodwell CE : Keeping an “ear” to the ground: seismic communication in elephants. *Physiology.* 2007;22(4):287–294. 10.1152/physiol.00008.2007 17699882

[ref-51] ell-Rodwell Osipov B Harvati K Nathena D : Sexual dimorphism of the bony labyrinth: a new age-independent method. *Am J Phys Anthropol.* 2013;151(2):290–301. 10.1002/ajpa.22279 23640711

[ref-52] Pantaleone J : Synchronization of metronomes. *Am J Phys.* 2002;70(10):992–1000. 10.1119/1.1501118

[ref-53] Peake WT Rosowski JJ Lynch TJ 3rd : Middle-ear transmission: acoustic versus ossicular coupling in cat and human. *Hear Res.* 1992;57(2):245–268. 10.1016/0378-5955(92)90155-g 1733916

[ref-54] Pinker S : The language instinct: How the mind creates language.Penguin UK.2003. Reference Source

[ref-55] Reilly J Peelle JE Garcia A : Linking somatic and symbolic representation in semantic memory: the dynamic multilevel reactivation framework. *Psychon Bull Rev.* 2016;23(4):1002–1014. 10.3758/s13423-015-0824-5 27294419 PMC5156531

[ref-56] Richards JC : Teaching Listening and Speaking From Theory to Practice.Cambridge, England: Cambridge university press.2008. Reference Source

[ref-57] Robinson H : Dualism.The Stanford Encyclopedia of Philosophy, Edward N. Zalta (ed.), Retrieved August 16, 2020,2019. Reference Source

[ref-58] Rost M : Teaching and researching: Listening.Routledge. Third edition.2016. Reference Source

[ref-59] Rost M Wilson JJ : Active listening.Routledge.2013. Reference Source

[ref-60] Sepulveda J : Fifty ways to teach them listening: Tips for ESL/EFL teachers.Portland, Oregon: Wayzgoose Press.2012. Reference Source

[ref-61] Seth AK : The Cybernetic Bayesian Brain. In Wiese, W. and Metzinger, T. K., editors, *Open MIND*. Frankfurt am Main, Germany: MIND Group.2014;9–24. 10.15502/9783958570108

[ref-62] Shapiro L : Embodied Cognition. New York: Routledge.2019. Reference Source

[ref-63] Shapiro L : Symbolism, embodied cognition, and the broader debate. *Symbols and embodiment: Debates on meaning and cognition*.2008;57–74. 10.1093/acprof:oso/9780199217274.001.0001

[ref-64] Shaw EA : The external ear. In *Auditory system*. Springer, Berlin, Heidelberg.1974;455–490. 10.1093/acprof:oso/9780199217274.001.0001

[ref-65] Spagnol S Geronazzo M Avanzini F : On the relation between pinna reflection patterns and head-related transfer function features. *IEEE Trans Audio Speech Lang Process.* 2012;21(3):508–519. 10.1109/TASL.2012.2227730

[ref-66] Skinner BF : Verbal behavior. New York: Appleton-Century-Crofts.1957. 10.1037/11256-000

[ref-67] Spivey M : The continuity of mind. Oxford University Press.2008. Reference Source

[ref-68] Strogatz S : Sync: The Emerging Science of Spontaneous Order. Hyperion Press.2003. Reference Source

[ref-69] Thelen E Smith LB : Dynamic systems theories. *Handbook of child psychology*.2007;1. 10.1002/9780470147658.chpsy0106

[ref-70] Tomasello M : Constructing a language: A usage-based theory of language acquisition. Cambridge, MA: Harvard University Press.2003. Reference Source

[ref-71] Tomasello M : The usage-based theory of language acquisition. In *The Cambridge handbook of child language*. Cambridge Univ. Press.2009;69–87. 10.1017/CBO9780511576164.005

[ref-72] Van den Bogaert T Carette E Wouters J : Sound source localization using hearing aids with microphones placed behind-the-ear, in-the-canal, and in-the-pinna. *Int J Audiol.* 2011;50(3):164–176. 10.3109/14992027.2010.537376 21208034

[ref-73] Van der Jeught S Dirckx JJ Aerts JR : Full-field thickness distribution of human tympanic membrane obtained with optical coherence tomography. *J Assoc Res Otolaryngol.* 2013;14(4):483–494. 10.1007/s10162-013-0394-z 23673509 PMC3705083

[ref-74] Vandergrift L Goh CC : Teaching and learning second language listening: Metacognition in action. Routledge.2012. Reference Source

[ref-75] Van Gelder T : What might cognition be if not computation? *J Philos.* 1995;92(7):345–381. 10.2307/2941061

[ref-76] Weir P : Dead Poets Society.Buena Vista Pictures Distribution.1989. Reference Source

[ref-77] Williams CJ Jacobs AM : The impact of otitis media on cognitive and educational outcomes. *Med J Aust.* 2009;191(S9):S69–S72. 10.5694/j.1326-5377.2009.tb02931.x 19883361

[ref-78] Wilson JJ : How to teach listening. Harlow, England: Pearson Longman.2008. Reference Source

[ref-79] Zwislocki JJ : The role of the external and middle ear in sound transmission. *The Nervous System Human Communication and its Disorders*.1975;3.

