# Supplementary material for: Mechanics of the Peripheral Auditory System: Foundations for Embodied Listening Using Dynamic Systems Theory and the Coupling Devices as a Metaphor
Source: F1000Res. 2021 Jul 28;10:193. Originally published 2021 Mar 8. [Version 2] doi: 10.12688/f1000research.51125.2 (PMC8258707; doi:10.12688/f1000research.51125.2)
Supplement: Peer review history

# Peer review: Reviewer response for version 1

**DOI:** 10.5256/f1000research.54249.r87992

**Published:** 2021-07-05

**Authors:** Brian J. Birdsell

**Affiliations:** 1 Center for Liberal Arts Development and Practices, Hirosaki University, Hirosaki, Japan

This paper explores embodied listening from an embodied cognition and dynamic systems perspective and how it may affect listening performance by foreign language learners. The paper is well-written and well-researched and contributes to both the fields of cognitive science and second language acquisition. A large portion of the paper is an in-depth analysis of the anatomy of the ear (particularly the external and mid-ear mechanisms) and the auditory process of making meaning from sounds. The author argues for using an elastomer coupling analogy, as opposed to the more commonplace Watt governor mechanism analogy, to illustrate the coupling between the different auditory mechanisms. In the end, the author then points out the wide variability of the ear apparatus among individuals and individual learners resulting in some having “golden” ears and others “tin” ears and this directly impacts academic outcomes in the target language.

 The paper successfully places the auditory process within a dynamic systems framework, but I would have liked to have seen the author elaborate in more detail “embodied listening” and how it fits within the larger framework of embodied cognition. Few would disagree that the auditory systems between individuals are highly variegated, but how does one proceed to measure this variance and how does this directly affect one’s cognitive performance on a given listening task? In short, can we actually measure this variability between those with “golden” as opposed to “tin” ears? Moreover, how does this “embodied listening” proposal fit into other theoretical approaches within the embodied cognition literature. For example, one possible avenue to further explore is an area of research called “body-specificity hypothesis” (Casasanto, 2009). ^1^ The main argument here is that body specific preferences, in this case, being either right or left-handed, has conceptual consequences in how one perceives abstract concepts like good and bad. For right handers, good is typically spatially viewed (through gestural movements) to be situated on the right side and left for bad. In contrast, for left handers this is the opposite. This suggests that the dominant hand affects how one conceives these evaluative abstract concepts. Specifically, how does auditory variation affect cognitive performance? Or how does auditory variation result in divergent interpretations from the same auditory stimuli?

 In regards to language learning, particularly a second language, the ideas presented in this paper bring up a number of important points. First, variation is the norm and likely is the result of exposure to the L2; age of exposure; but as presented in this paper, physiological characteristics of the auditory system. How can teachers know whether a breakdown in listening should be contributed to a lack of exposure to the sounds of that language or some possible physiological variation with the auditory channel? Some practical guidelines for language teachers would be beneficial here. Secondly, this paper also seems to support the theoretical view that all communication is “good enough” (Ferreira, Bailey, & Ferraro, 2002). ^2^  and instead of viewing concepts and categories as stable constructs, it is more fruitful to view them as dynamic and ad hoc (Cassanto & Lupyan, 2015) ^3^ and highly variable dependent on context and individual variation, both at the experiential level, but also physiological level.

Is the topic of the opinion article discussed accurately in the context of the current literature?

Yes

Are arguments sufficiently supported by evidence from the published literature?

Yes

Are all factual statements correct and adequately supported by citations?

Yes

Are the conclusions drawn balanced and justified on the basis of the presented arguments?

Yes

Reviewer Expertise:

Embodied cognition, creativity, and metaphor theories

I confirm that I have read this submission and believe that I have an appropriate level of expertise to confirm that it is of an acceptable scientific standard.

## References

[1] : Embodiment of abstract concepts: good and bad in right- and left-handers. *J Exp Psychol Gen* .2009;138(3) : 10.1037/a0015854 351-67 10.1037/a0015854 19653795

[2] : Good-Enough Representations in Language Comprehension. *Current Directions in Psychological Science* .2002;11(1) : 10.1111/1467-8721.00158 11-15 10.1111/1467-8721.00158

[3] : All concepts are ad hoc concepts. The conceptual mind: New directions in the study of concepts. *The MIT Press* .2015;543-566

# Response

**Published:** 2021-07-26

**Authors:** Bruno Jactat

**Affiliations:** University of Tsukuba, Japan

Professor Birdsell, thank you for your concise and clear summary of my work as well as the valuable feedback and pointers you have kindly provided.

 The notion of “embodied listening” indeed remains to be developed as this paper focused principally on hearing rather than listening. As another reviewer suggested, the title being misleading, it needs to be revisited. To that effect, the article now comes with a new title: “Mechanics of the Peripheral Auditory System: Foundations for Embodied Listening Using Dynamic Systems Theory and the Coupling Devices as a Metaphor.”

 The sequel paper will definitively delve into “embodied listening” in more detail. I appreciate your suggestion to frame it into the larger topic of embodied cognition, a piece of advice I will follow on in the next paper.

 Casanto’s “body-specificity hypothesis” is one of those frameworks that can likewise be explored in auditory processing. Research over decades show that humans have a side-dominant ear, just like we have a dominant hand and eye. This in turn does guide behaviors and has practical implications in the classroom. I will actually be addressing these issues, such as how the right ear generally makes more sense of language than the left (known as the Right Ear Advantage—REA). Your questions regarding how auditory perception is influenced by body specific preferences will be developed when considering listening, as ear-preference is linked with the individualized neuro-architecture of brain hemispheres.

 Practical guidelines will be offered to teachers once those issues addressed. As an example, simply asking students in class to hold their smartphones to their ear, usually points to their dominant ear. By asking students to seat themselves in the classroom so that their dominant ear is turned toward the teacher is thought to help understanding. Teachers should also place their own desk slightly to the left side of the room when facing the class as there are statistically more right-eared people than left-eared. Too often desk position is aleatory and simply placed opposite the classroom entrance. Similar guidelines will be drawn out in a sequel paper. Because there was a paragraph right before the “Topic Overview” section that didn’t hold its promise of providing such cues, I have taken it out altogether.

 I have looked at the references you suggested regarding ‘Good-Enough Representations’ and will include these views in the future, as they reflect the top-down processes in listening that will be discussed.

 Let me reiterate my sincere gratefulness for your review and the stimulating questions that you have brought up. They will definitively help guide my research and reflections to come.

# Peer review: Reviewer response for version 1

**DOI:** 10.5256/f1000research.54249.r81098

**Published:** 2021-04-06

**Authors:** Curtis Kelly

**Affiliations:** 1 Faculty of Business and Commerce, Kansai University, Osaka, Japan; 2 JALT BRAIN SIG, Tokyo, Japan

**Pros & cons:**

 +  like the way that this is written for teachers as well as academics, as I think most papers should be.

 + Finally, someone looking at something specific (listening) from the bigger view (systems).

 ? Is listening the right term?  Or is it hearing?

 - If space is a problem, I am not sure we need that much history of coupling, initial parts could be condensed.  Then again, the main purpose of this paper seems to be making a metaphor that helps us understand the mechanical and neural mechanisms of listening, which is done with coupling.  Therefore, I think the paper title would better prepare the reader if it were something like “The Mechanics of Embodied Auditory Perception: Using the Coupling Devices as a Metaphor.

 + I like the way the paper posits the steps or auditory processing from signal to action, coving both mechanical and neural activity.

 - I’d like even just a little bit on the processing part because theories like predictive processing say that auditory sensation is passed down from higher cortical regions rather than just processed through input, ie, transmission is two-way, but it seems this is the next paper.

 + In neuroscience, we are finding more and more that the brain builds advanced concepts from real life experiences which are used metaphorically. That making such a metaphor is the purpose of this paper is kind of exciting, and something we will see more of in the future.

 Even though a lit review, In the section just before “Coupling,” I’d like to see a couple of paragraphs outlining the mechanics of listening/hearing, not just embodiment, and then proposing that we might get a better understanding of that process by looking at coupling.

 +Writing quality is superb.

Is the topic of the opinion article discussed accurately in the context of the current literature?

Yes

Are arguments sufficiently supported by evidence from the published literature?

Yes

Are all factual statements correct and adequately supported by citations?

Yes

Are the conclusions drawn balanced and justified on the basis of the presented arguments?

Yes

Reviewer Expertise:

neuroscience of learning, predictive processing and embodiment,

I confirm that I have read this submission and believe that I have an appropriate level of expertise to confirm that it is of an acceptable scientific standard.

# Response

**Published:** 2021-07-26

**Authors:** Bruno Jactat

**Affiliations:** University of Tsukuba, Japan

Professor Kelly, my sincere gratitude goes to you for taking the time to review this long article.

 The length of the text might indeed have been shortened or better yet made into two shorter articles. My purpose here was 1) to revise the current metaphor used to illustrate the notion of ‘coupling’ within Dynamic Systems Theory, and 2) to use the updated metaphor to underline how variegated coupling appears in hearing and listening, and by extension highlight the uniqueness of individuals, how no two pair of ears are identical. Therefore the bulk of the text which could be considered as two endeavors put into one.

 Since this article serves as a foundation to sequel papers—that will delve more into the listening processes you aptly describe as comprising functions such as ‘predictive processing’ (“…auditory sensation is passed down from higher cortical regions…”)—I have made only minor changes to slightly shorten the text (two paragraphs have been deleted).

 Moreover, your comment about revisiting the title hits the nail on the head: Indeed, listening has been barely encroached on within this paper as the contents rather lay the foundations of hearing mechanisms. I have accordingly changed the title to:

 “Mechanics of the Peripheral Auditory System: Foundations for Embodied Listening Using Dynamic Systems Theory and the Coupling Devices as a Metaphor.”

 Your suggestion of adding “a couple of paragraphs outlining the mechanics of listening/hearing, not just embodiment” would definitively  help the reader. As I have already succinctly described the difference in the paragraph right before the ‘Dynamics system theory’ section, and not willing to further lengthen the text, I will make good use of your suggestion in the upcoming paper and furthermore develop the distinction there.

 Once again, thank you very much for your positive comments which encourage me to pursue my research and reflections on these topics.
